# Curve-on-curve technique does not improve tibial coverage in total knee arthroplasty in comparison to tibial tuberosity technique with use of anatomical implants: randomized controlled trial

**DOI:** 10.1007/s00402-023-04857-3

**Published:** 2023-03-31

**Authors:** Bartosz M. Maciąg, Tomasz Kordyaczny, Krystian Żarnovsky, Martyna Budzińska, Dawid Jegierski, Marcin Łapiński, Grzegorz J. Maciąg, Artur Stolarczyk

**Affiliations:** Department of Orthopedics and Rehabilitation, Medical University of Warsaw, Międzyleski Specialist Hospital, 2 Bursztynowa St., 04-749 Warsaw, Poland

**Keywords:** Total knee arthroplasty, Anatomic design, Tibial rotation, Coverage

## Abstract

**Introduction:**

During the last years, main attention while performing total knee replacement was paid to femoral component alignment; however, there is still lack of studies concerning tibial baseplate rotational alignment, especially in terms of anatomical designs of knee prosthesis. Some recent studies proved that tibial baseplate malrotation might be a cause of knee pain and patients’ dissatisfaction. The aim of this study was to compare tibial component rotation and its coverage on the tibial plateau achieved with curve-on-curve and tibial tuberosity techniques (t-t technique) with use of anatomic knee designs with asymmetric tibial baseplate.

**Materials and methods:**

A total of 88 patients were randomly assigned in a 1:1 ratio to undergo total knee arthroplasty with use of the PERSONA PS (Zimmer Biomet) knee design with an asymmetric baseplate. The rotation of the tibial component was assessed and performed with two different techniques: curve-on-curve technique and tibial tuberosity technique. Tibial component rotation was measured on computed tomography (CT) scans using the method suggested by Benazzo et al. and designed for asymmetrical implants. For the measurement of the tibial bone coverage, the component surface area was outlined and measured on a proper CT section, then the tibial cut surface area was outlined and measured on a section just below the cement level. Pre- and post-operative range of motion was measured by another independent researcher 12 months post-operatively during follow-up visit.

**Results:**

There was a statistically significant difference between both groups in median value of tibial rotation angle: 7° (interquartile range (IQR) = 0–12) in curve-on-curve technique group vs 2° (IQR-1–7) in tibial tuberosity technique group, probability value (*p*) = 0.0041, with values above 0 meaning external rotation of the component. There was no statistically significant difference between both groups in terms of range of motion (ROM) with average values of 124.3° ± 13.0° for curve-on-curve technique and 125.6° ± 12.8° for t-t technique with *p* = 0.45. There was a statistically insignificant difference between both groups in terms of coverage percentage in slight favor for curve-on-curve technique (85.9 ± 4.2 vs 84.5 ± 4.8, *p* = 0.17).

**Conclusion:**

In this study, no difference between the groups in terms of tibial bone coverage and range of motion was proved, even though both techniques differed significantly with values of tibial rotation. Future studies should be focused on influence of specific values of tibial rotation on patient-reported outcomes and survivorship of anatomic knee implants.

## Introduction

Total knee replacement (TKR) is a surgical procedure with a relatively high rate of success, low rate of complications, and a growing expected survivorship. Even so, it is estimated that the percentage of dissatisfied patients after TKR might be as much as 20% [1, 2]. One of the reasons for occurrence of complications such as residual pain, instability or implant loosening is improper component placement in which rotational alignment is one of the key aspects. During the last years, main attention was paid to femoral component alignment [3–5]. It resulted in development of surgical technique and tools along with establishment of new surgical landmarks for proper implant placement. However, there is still a lack of studies concerning tibial baseplate rotational alignment, especially in terms of anatomical designs of knee prosthesis [6, 7]. In recent studies, it was proved that tibial malrotation might be a cause of knee pain and patients’ dissatisfaction. “Curve-on-curve” and “range-of-motion” techniques were described in the literature to optimize the tibial baseplate rotation and, therefore, improve post-operative range of motion, bone coverage, and patellar tracking [8, 9]. In addition, several anatomic landmarks were established such as medial border of the tibial tuberosity, the medial third of the tibial tuberosity, the anterior tibial crest, the posterior tibial condylar line, the space in the middle of malleoli, and the second ray of the foot. Anatomical baseplates were developed to more accurately reproduce the native contour of tibial plateau [8, 10–12]. That is important as even minor overhang of the tibial component might cause pain and malfunction of the joint. There are also studies indicating that underhang in particular zones of tibial plateau might be the risk factor of tibial bone resorption process, which might, in the future, lead to aseptic loosening of components. The aim of this study was to compare tibial component rotation and its coverage on the tibial plateau achieved with curve-on-curve and tibial tuberosity techniques with use of anatomic knee designs with asymmetric tibial baseplate [13–15].

## Materials and methods

### Selection of the study cohort

The authors followed the guidelines for reporting parallel-groups, randomized, and controlled trials. From January 2021 to February 2022, 94 patients were enrolled in the study. They were qualified by a single experienced surgeon for TKR and were randomly divided into two groups, depending on the technique later used to establish tibial component rotation. Inclusion criteria were: primary knee osteoarthritis, varus or neutral lower limb alignment (0° to 15° varus), preoperative Insall-Salvati ratio between 0.8 and 1.2. Exclusion criteria included secondary knee osteoarthritis, any previous lower limb surgery or ligamentous injury, patients with severe deformity with > 15° of varus, valgus or fixed flexion deformity. All participants received on-label use of PERSONA posterior-stabilized implants without patella resurfacing. Patients were randomly assigned in a 1:1 ratio to undergo total knee arthroplasty with use of PERSONA PS (Zimmer Biomet) knee design with asymmetric baseplate. The rotation of the tibial component was performed with two different techniques.

### Randomization

Randomization process was conducted with a computer software based on the age, BMI, and the operated side. During the whole treatment and follow-up process, only the surgeon was aware, which technique was used. Researchers measuring rotation, range of motion, and tibial baseplate coverage were unaware of the technique used in particular cases.

### Surgical technique

All surgeries were performed in a level III academic hospital with use of a tourniquet (average time of 80 min) and post-operative closed suction drainage left for at least 12 h. All surgeries were initiated with the use of standard midline incision and medial parapatellar arthrotomy. Tibial cuts were done first using extramedullary alignment jigs. These were made perpendicular to the long axis of the tibia with a posterior slope between 0° and 7°, adjusted to the native posterior tibial slope. The femur was prepared using intramedullary alignment with a valgus angle between 5° and 7°, according to valgus correction angle measured on long-leg standing radiograph. External rotation cuts were done with the posterior condylar axis perpendicular to the transepicondylar line. Femoral bone cuts were made in the sequence as recommended by the surgical protocol. After removal of posterior and peripheral osteophytes, soft-tissue balance was assessed using the FUZION dynamic balancer. Flexion and extension gaps were balanced. No patella resurfacing was performed. All components were implanted with the use of cement.

### Curve-on-curve technique

After all bone cuts, the tibial template was placed onto the plateau and its positioning was adjusted to the posterolateral curve of the tibia and rotation was adjusted to cover as much of the plateau as possible. All further surgical steps were done according to the manufacturer guide.

### Tibial-tuberosity technique

After all bone cuts, the tibial template was placed onto the plateau with rotation adjusted for the middle of tibial tuberosity. After estimating proper rotation, the size of the component was picked to fit the plateau without overhang. All further surgical steps were done according to the manufacturer’s guide.

### Post-operative care

The post-operative protocol included chemical and mechanical thromboprophylaxis unless specifically contraindicated. All patients received one dose of parenteral antibiotics at the induction of anesthesia and two further doses post-operatively.

Flexion and extension exercises of the ankle and isometric quadriceps contraction exercises were started on the first post-operative day, with full weight-bearing within pain tolerance. The duration of the exercises was 40 min to 1 h three times per day. All exercises were done bedside without using additional rehabilitation equipment. The aim of mobilization with a physiotherapist was to obtain flexion of the knee of at least 90°. Other methods of mobilization included using a walker or walking with crutches by the third day post-op. The average length of stay in the hospital was 3.3 days (3–4).

### Primary outcome

#### Radiographic evaluation

**Fig. 1:**
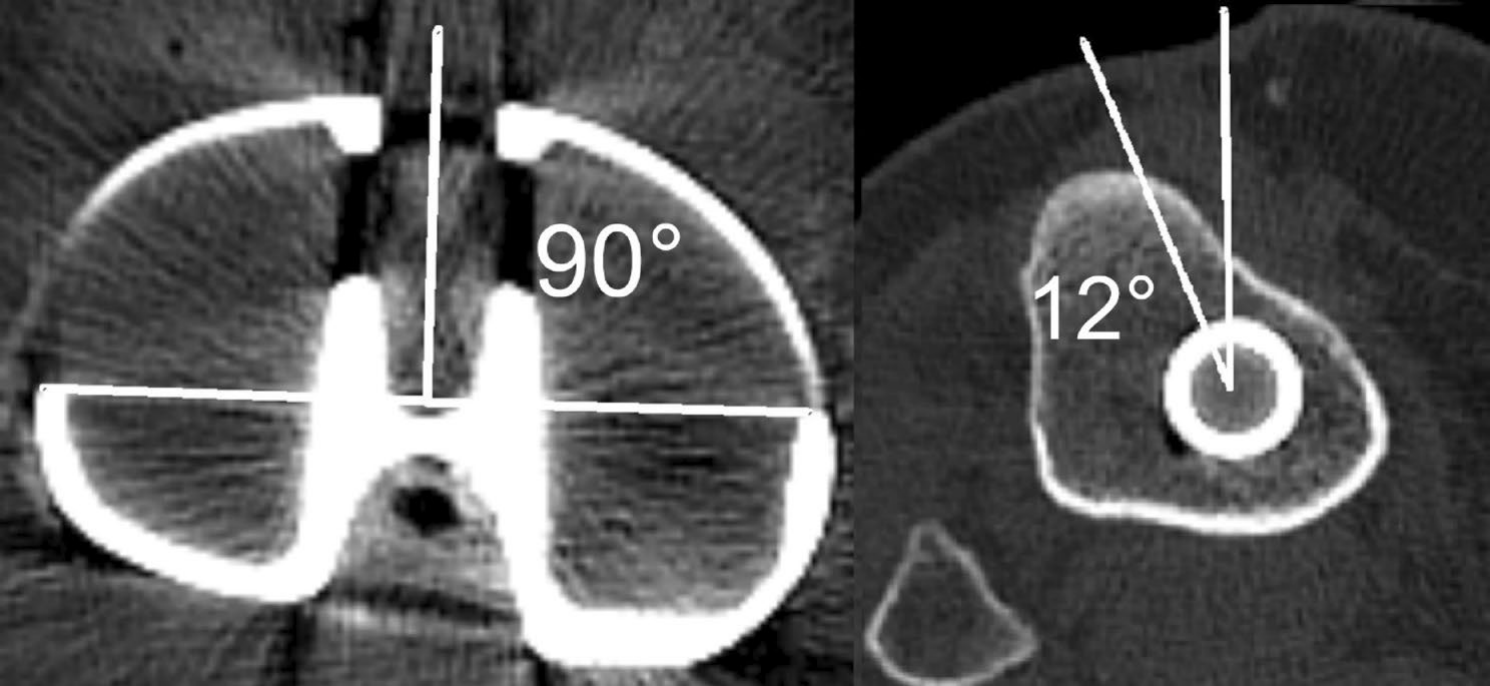
Tibial component rotation measurement, in this case – 12° of internal rotation

**Fig. 2:**
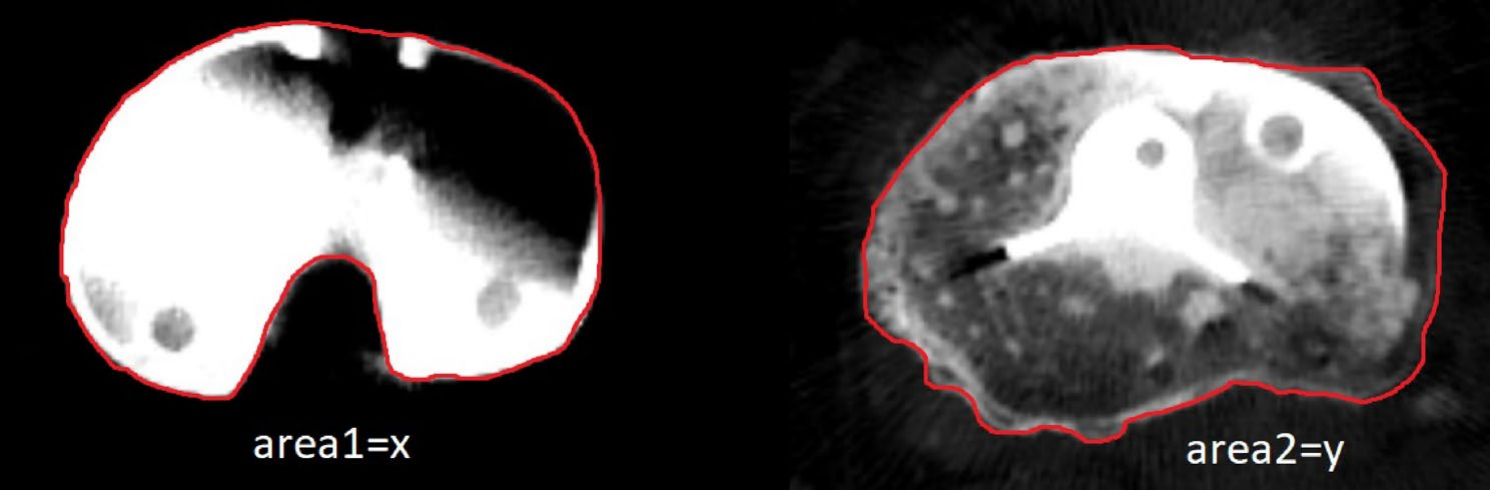
Tibial coverage measurement. Area1 represents tibial component surface, area2 represents tibial cut surface just below the cement level

The computed tomography images were measured and reviewed by two experienced orthopedic surgeons, who did not take part in the surgery or further research. Any disagreement between them was solved by the senior author of this study. All knees underwent CT evaluation in the post-operative period using Philips Incisive CT (Philips Healthcare, Cleveland, OH, USA). Each patient signed an informed consent for the CT examination. Both rotation and coverage measurements were performed using the INFINITT program by two independent orthopedic surgeons, who did not take part in any other activity associated with this study. Tibial component rotation was measured on CT scans using the method suggested by Benazzo et al. [16] and designed for asymmetrical implants (Fig. 1). The tangent line to two posterior wings was drawn and the second line determining the anterior–posterior (AP) axis perpendicular to the first line and passing through the center of the anterior hole. The third line, starting from the cross-point and passing through the medial one third of tibial tuberosity (TT), defined the rotation angle with the AP axis. Depending on the angle location (medially, laterally) in relation to the AP axis, the rotation was described, respectively, as internal or external. For the measurement of the tibial bone coverage, the component surface area was outlined and measured on a proper CT section, then the tibial cut surface area was outlined and measured on a section just below the cement level. Concurrently, the overcoverage or undercoverage site was noted (Fig. 2).

### Secondary outcomes

Pre- and post-operative range of motion was measured by another independent researcher 12 months post-operatively during follow-up visit.

### Statistical analysis

Power analysis was conducted using G*Power version 3.1.9.4 (Faul et al. 2007) to determine the minimum sample size required to test the study hypothesis. The effect size was calculated as Cohen’s *d*-value equal to 0.615 based on results obtained from the first 28 patients enrolled in the study. With *α*-value set at 0.05 and 80% power, sample size was estimated as *N* = 90 using Mann–Whitney *U* test.

Statistical analysis of results was performed. Due to significant deviation from normality of distribution for all continuous variables, Mann–Whitney *U* test was used and for categorical variables, Fisher’s exact test was used. All comparisons were performed between independent groups. An *α*-value of 0.05 was used to determine statistical significance of all the analyses. All statistical analyses were conducted using SAS software, Version 9.4 for Windows (SAS Institute Inc., NC, USA).

## Results

### Participants

Eighty-eight participants were eligible for final assessment during follow-up (93.6%). One patient from the initial group underwent manipulation under anesthesia 5 weeks post-operatively, due to arthrofibrosis, another one was diagnosed with COVID-19 in the post-operative period and did not undergo proper rehabilitation. Two of the participants from both groups did not come for the follow-up visit. Baseline characteristics of participants are depicted in Table 1.

**Table 1:** Baseline characteristics of the study group

	Curve-on-curve technique (*n* = 46)	t-t technique (*n* = 42)	Total (*n* = 88)	*p* value
Age (years)	68.2 ± 7.61	69.93 ± 7.86	mean: 68.1 ± 7.7	0.9
BMI	28.9 ± 5.5	29.2 ± 5.1	29.0 ± 5.9	0.9
Female/male	31/15	29/13	60/28	1
Left / right knee	23/23	14/28	37/51	0.1

### Measurements

There was a statistically significant difference between both groups in median value of tibial rotation angle: 7° (IQR = 0–12) in curve-on-curve technique group vs 2° (IQR-1–7) in tibial tuberosity technique group, *p* = 0.0041, with values above 0 meaning external rotation of the component. There was no statistically significant difference between both groups in terms of ROM with average values of 124.3 ± 13.0 for curve-on-curve technique and 125.6 ± 12.8 for t-t technique with *p* = 0.45. There was a statistically insignificant difference between both groups in terms of coverage percentage in slight favor for curve-on-curve technique (85.9 ± 4.2 vs 84.5 ± 4.8, *p* = 0.17). Results are depicted in Table 2.

**Table 2:** Clinical and radiological outcomes

	Curve-on-curve technique	t-t technique	*p* value
Median value of tibial rotation angle (°)	7 (IQR = 0–12)	2 (IQR = 1–7)	0.0041
Maximum extension (°)	2.07 ± 4.16	1.67 ± 4.37	0.6
Maximum flexion (°)	126.41 ± 11.43	127.26 ± 10.01	0.6
ROM (°)	124.3 ± 13.0	125.6 ± 12.8	0.45
Tibial bone coverage (%)	85.9 ± 4.2	84.5 ± 4.8	0.17

## Discussion

To our best knowledge, this is the first randomized controlled study to assess tibial rotation and tibial baseplate coverage with the use of anatomic implants between these two surgical techniques. The most important findings of this study are significant differences in tibial rotation between techniques with less externally rotated implants in t-t technique and insignificant difference in tibial plateau coverage. However, these factors did not affect post-operative range of motion.

### Tibial rotation

There were many comparative studies analyzing values of tibial baseplate rotation between various techniques. In the most recent ones, there were no statistically significant differences neither in range of motion nor patient-reported outcome between surgical techniques [17–21]. However, the majority of these studies were performed using symmetric knee designs. There is a limited number of papers, which analyzed these outcomes using asymmetric anatomical implants. In the study by Indelli et al. [19], authors compared tibial rotation between symmetric and asymmetric knee designs using curve-on-curve technique in relation to surgical transepicondylar line. With the use of asymmetric one, there were significantly more external values of baseplate rotation, with 20% of symmetric baseplates being rotated internally, while none of asymmetric ones were. This can only partly correspond with results of our study as the benchmark for assessing rotation was different in both studies. Such results might favor the t-t technique as stated in the studies by Abdelnasser et al. and Bell et al. [22, 23], where authors stated that internal rotation of the tibial component might be the reason of a painful knee after the surgery as well as an extension deficit. On the other hand, in the review by Osano et al., authors stated that excessive external rotation of the tibial component might lower the survivorship of the polyethylene insert [24]. t-t technique must be performed with caution to tibial bowing, as stated in the study by Palanisami et al. [25] more than 3° of extra-articular deformation in varus knees might result in excessive lateralization of the tibial component.

### Tibial coverage

It is believed that at least 75% of tibial coverage by the baseplate is needed to obtain adequate fixation [26]. In the study by Meier et al. [27], authors measured tibial coverage of several components comparing symmetrical and asymmetrical implants. Adjusting coverage to proper rotation to 1⁄3 of the tibial tuberosity PERSONA knee design (asymmetrical) provided the best bone coverage of the tibia. In our study, high tibial coverage was obtained regardless of used technique. In the study by Martin et al. [28], authors simulated tibial coverage on 30 specimens in CT with the use of asymmetric implants. It resulted in significantly less malrotated internal components, as it was easier to estimate the relation between tibial rotation and coverage. Results of this study are not confirmed by another study by Shao et al. [29] in which the authors stated that maximizing tibial plateau coverage does not necessarily result in implant malrotation. In our study, choice of technique had a significant impact on the value of tibial baseplate rotation. Clary et al. [30] concluded in their study that setting rotational alignment by maximizing coverage should be avoided for all tibial base designs because of the risk of excessive internal rotation. On the other hand, in the study by Clary et al. [30], authors did not find significant superiority in terms of tibial coverage in favor of neither symmetric nor asymmetric implants. What they found was that with the maximizing coverage technique that they used, asymmetric implants provided more internal rotation of the component. Lützner et al. [20] proposed a so-called “safe zone” that allows surgeons to optimize the tibial coverage with use of two landmarks to avoid patellar maltracking. One of the weak points of this technique is that it is difficult to precisely adjust the rotation to the medial 1⁄3 of the tibial tubercle during the surgery.

### Limitations of the study

Even though this is a high-quality randomized controlled study with use of single knee design implants, one surgeon performing all surgeries and little loss of participants to follow-up, it certainly has some limitations. First of all, the 12-months follow-up is a relatively short observation time and second, no assessment of patient-reported outcome was collected apart from range of motion.

## Conclusion

Both techniques have their strong and weak points and with use of anatomic implants, it seems that both are useful in performing total knee arthroplasty. In this particular study, no differences in terms of tibial bone coverage and range of motion were proved, even though both techniques differed significantly with values of tibial rotation.

## Data Availability

The data that support the findings of this study are available from the corresponding author, BMM, upon reasonable request.
